# Editorial: ‘Divided or united': strengthening social cohesion for well-being and prosperity

**DOI:** 10.3389/fpsyg.2023.1278832

**Published:** 2023-11-10

**Authors:** Nima Orazani, Diana Cárdenas, Katherine J. Reynolds, Rodolfo Mendoza-Denton

**Affiliations:** ^1^Department of Psychology, Carleton University, Ottawa, ON, Canada; ^2^Department of Psychology, University of Montreal, Montreal, QC, Canada; ^3^Department of Psychology, The Australian National University, Canberra, ACT, Australia; ^4^Department of Psychology, University of Melbourne, Melbourne, VIC, Australia; ^5^Department of Psychology, University of California, Berkeley, Berkeley, CA, United States

**Keywords:** social cohesion, social identity, multiculturalism, community resilience, resilience, contact theory

Social cohesion is a construct of wide interest across a range of disciplines (e.g., economics, sociology, political science, psychology) as well as government and non-government agencies. Both the Organization for Economic Cooperation and Development (OECD) and the Council of Europe have published extensive literature on social, political, cultural and economic factors that threaten social cohesion (Jeannotte et al., 2002). These diverse perspectives have also generated various (and, at times, confusing) definitions, measurements and practical interventions aimed at strengthening social cohesion, which is often considered a “quasi” or “hybrid” concept. Most consensus exists where social cohesion refers to a pattern of relationships and behaviors that include trust, a sense of belonging, willingness to help others (including across ethnic and diversity groups) and confidence in (the legitimacy of) political and social institutions (e.g., Chan et al., 2006; Dragolov et al., 2016).

Higher social cohesion brings better health and wellbeing outcomes (Kawachi and Berkman, 2000), less loneliness (O'Donnell et al., 2022; Hajek et al., 2023) and underpins a community's ability to act to solve collective problems (Jewett et al., 2021). Despite its importance, there is evidence across many countries that this “social glue” is fragile and at risk. Thus, there is an urgent need for large-scale, theory-driven, and empirically tested solutions to maintain and strengthen it.

One aim of this Research Topic “‘*Divided or united': strengthening social cohesion for well-being and prosperity*” is to refocus attention on social cohesion as an area of evidence-based enquiry which can be utilized to transform communities. Along these lines, a recent multi-year, interdisciplinary research project at the Australian National University, Canberra, was designed to develop and advance renewed rigor surrounding social cohesion. There is a growing body of work on social cohesion and COVID-19 (Cárdenas et al., 2021, 2023; O'Donnell et al., 2022), systematic reviews (Orazani et al., 2023a,b), a new measurement scale (Orazani et al., 2023a,b) and social enterprises as cohesion-building entities (Qureshi et al., 2021). Key insights should be able to be transposed to new settings and be upscaled.

## Social cohesion: a social psychological approach

The Research Topic also aims to draw scholars and policy makers' attention to well-researched, yet largely overlooked, social psychological theories. Areas that have not been fully embraced include contact theory (e.g., Darling-Hammond et al., 2021; White et al., 2021) and social identity processes, especially with respect to discrimination, prejudice, and intergroup conflict and co-operation (Tajfel and Turner, 1979; Turner et al., 1987; Turner and Reynolds, 2012). A central idea is that people can define themselves in terms of their social identity as a group member (“we” and “us”) which emerges in comparison to other groups. The more people identify with their group (e.g., their nation, sports team), the more likely it is for them to (a) engage in behaviors that better the group—including following ingroup norms and advancing group goals (Zhou et al., 2023); (b) attenuating trust (Cruwys et al., 2021), help and connect with ingroups, and (c) seek to resolve group disagreements in constructive ways through mutual influence (Turner, 1991). In fact, social identity can be an important driver of contact (White et al., 2021) and group cohesion (Hogg et al., 1995) and other indicators of group functioning (Haslam et al., 2003; Haslam, 2004). Critically, the concept of cohesion can apply to any group (e.g., sport teams, neighborhood, work team) and can be informed by other dimensions of group psychology including the role of leadership and collective action (Orazani et al., 2023a,b). These insights open up a new horizon for “social” cohesion research, one in which group processes are at the heart of the glue that binds us.

Many of the six articles of this Research Topic draw on these group psychology approaches or related ideas, and all make an important contribution and offer a path forward in strengthening social cohesion. For example, Cruwys et al. found that Neighbor Day, a grass-root, community-led intervention, promoted strong neighborhood identification which protected community members against the negative mental health effects of lockdown. Vine and Greenwood evaluated the benefits of community solidarity initiatives (CSIs) where displaced people and residents/nationals engage in contact activities. Cross-group friendships from CSIs predicted stronger collective action intentions. Dierckx et al. found that procedural fairness regarding cultural decisions leads to positive outcomes, as majority and minority members react positively to fair treatment of others—a key ingredient in social cohesion. Eldor et al. focused on schools as group-based environments with norms, values and beliefs that can be cohesion-promoting. Using their newly developed scale, they found that an egalitarian school environment was associated with lower extremist intentions and radicalization. Hartz et al. demonstrated the resilience of social cohesion among youth, citizens of active age, and the elderly when they faced the current pandemic. Lastly, Van Assche et al. contest the assumption that (objective and perceived) diversity is necessarily negative for cohesion, highlighting the key role of segregation.

This Research Topic includes diverse samples (Australia, Belgium, Ireland, Germany, Norway, The Netherlands) and groups including the local community (Cruwys et al.; Vine and and Greenwood), minority and majority members (Dierckx et al.; Van Assche et al.) and young people (Eldor et al.; Hartz et al.). Diverse methodologies are also utilized including field studies (Cruwys et al.; Vine and Greenwood), laboratory experiments (Dierckx et al.), new scale development (Eldor et al.) and survey research (Hartz et al.; Van Assche et al.). Overall, this body of work highlights the huge contribution of high-quality research on social cohesion and the promise of large-scale interventions to build social cohesion to protect people's wellbeing and prosperity, especially for those who are most vulnerable and experiencing crises. Our hope is that the work outlined in this Research Topic will be an accelerator for a greater number of evidence-based and scalable initiatives to strengthen the relational infrastructure necessary to thrive together.

## Author contributions

NO: Conceptualization, Writing—original draft, Writing—review & editing. DC: Conceptualization, Writing—original draft, Writing—review & editing. KR: Conceptualization, Writing—original draft, Writing—review & editing. RM-D: Writing—original draft.
